# Factors influencing consumers’ willingness to accept service robots: Based on online reviews of Chinese hotels

**DOI:** 10.3389/fpsyg.2022.1016579

**Published:** 2022-10-11

**Authors:** Cheng Chang, Bingjia Shao, Yong Li, Yong Zhang

**Affiliations:** School of Economics and Business Administration, Chongqing University, Chongqing, China

**Keywords:** service robot, human–robot interaction, online review, service robot integration willingness scale, content analysis

## Abstract

The application of robots in service industry is increasing. Compared with related studies in other countries, the research on users’ acceptance of mid-range and high-range hotel service robots in China is preliminary. Based on the interaction between Chinese consumers and hotel service robots, this study explored the factors that influence consumers’ willingness to accept robots in human–robot interaction. According to the service robot integration willingness scale (performance efficacy, intrinsic motivation, anthropomorphism, social influence, facilitating conditions, and emotion), this study conducted content analysis and sentiment analysis on 4,107 online reviews from 68 mid-range and high-range hotels in Qunar. The results showed that users’ overall evaluation of robot service in mid-range and high-range hotels is positive. The most frequently mentioned dimension by users is performance efficacy, followed by intrinsic motivation, anthropomorphism, and emotion, finally, the facilitating conditions, the five dimensions have positive impact on users’ evaluation of service robots; the influence of social influence on human–robot interaction evaluation has not been found. This study supplements the research on service robot and provides a reference for hotel managers to make decisions.

## Introduction

As a typical frontline application of business scenarios, service robots can reduce the labor cost of enterprises and bring new experiences to customers. Service robot can be defined as an autonomous and adaptable interface based on the system, which can interact, communicate, and provide services to the customers of the organization (Wirtz et al., 2018). Robot application in hotels (Ivanov et al., 2018a,b; Qiu et al., 2020; Yu, 2020; Ab Rahman et al., 2022), tourism (Tung and Law, 2017), catering (Cha, 2020), and other service areas are gradually emerging. van Doorn et al. (2017) predicted that by 2025, service robot technology would be applied to more service scenarios, and the outbreak of COVID-19 further accelerated the robot’s expansion to other fields, such as express delivery, housekeeping, security, etc. (Go et al., 2020).

Compared with other industries, the high touch of hotel services makes service robots have broad application prospects in hotels, scholars have revealed advantages of hotel service robots. Service robots can promote novel, interesting and flexible interaction with customers, thus benefiting hotels (de Kervenoael et al., 2020). Human–robot interaction experience is the decisive factor that affects the consumer’s emotional experience of hotel brands (Hwang and Seo, 2016). The presence of texts related to service robot in online reviews influences positively electronic word-of-mouth valence (Mariani and Borghi, 2021). Generation Z customers consider that robots reduce contagion risk at hotels (Romero and Lado, 2021). Although robot technology is particularly important in hotels, the related research topics of human–robot interaction are still preliminary (Ivanov et al., 2018b; Schreibelmayr and Mara, 2022), in which consumers’ acceptance and willingness to use service robots is the focus.

As a prerequisite for consumers to keep using robots, the research on consumers’ willingness to accept hotel service robots is increasing gradually. Although the methods and data used in related research are more diversified and the theories are gradually improved, there are always conflicts on key research conclusions. It is mainly reflected in three points. First, the conclusions of factors influencing acceptance are not uniform. For instance, some scholars found anthropomorphism negatively affects consumers’ willingness to accept robots and their experience (Gursoy et al., 2019; Lu et al., 2019; Roy et al., 2020; Yu, 2020), while Qiu et al. (2020) found anthropomorphism positively affected the rapport between users and robots and the service hospitality experience. Second, although high-range hotels introduced service robots earlier, there is no consistent conclusion on the applicability. In high-range hotels, previous studies found robot services reduce consumers’ behavioral experience compared with employee services (Chan and Tung, 2019), users’ perceived service interaction quality and physical service environment evaluation of robot services were lower than those provided by employees, and there was no significant difference in the quality of results (Choi et al., 2020); however, Fuentes-Moraleda et al. (2020) found that users’ overall acceptance was high and the evaluation of robot services is generally positive. Third, the potential use of service robots in the Chinese hotel industry is huge, and there are already many application practices at present, but there is still few research concerned about Chinese consumers’ acceptance of hotel service robots. Previous research found factors influencing consumers’ acceptance exist differences between countries in multinational samples (Fuentes-Moraleda et al., 2020; Choi et al., 2021). However, those studies collected few samples of Chinese consumers and lack detailed analysis to reflect the elaborate factors that affect Chinese users’ acceptance. Whether the extent conclusions can be extended to the hotel service robots in China remains to be discussed.

Considering the above three gaps, it is necessary to further study the factors affecting the acceptance of service robots and the applicability of service robots in Chinese hotels. The article is divided into five parts. The second part introduces the literature review of this article, the third part introduces the research methods, the fourth part is about the research results, and the fifth part is the conclusion, limitations, and future directions.

## Literature review

### Service robots and consumer acceptance

At first, the research on human–robot interaction acceptance and intention to use robot was mainly based on the Technology Acceptance Model (TAM). After that, the widely used models include USUS (based on usability, social acceptance, user experience, and social influence) (Weiss et al., 2009), Godspeed questionnaire (based on anthropomorphism, animacy, likeability, perceived Intelligence, and perceived safety) (Bartneck et al., 2009) and UTAUT model (Venkatesh et al., 2012). With the gradual application of robots in the service field, exploratory studies on the acceptance and adoption of service robots have begun to appear (such as Park and del Pobil, 2012, 2013), the research methods are mainly questionnaires or experiments. As the advances of research, new influencing dimensions and factors are constantly identified and integrated. For example, Wirtz et al. (2018) compared the differences among service robots, self-service technologies, and employees, and put forward a conceptual model—the service robot acceptance model (sRAM) building on the TAM. It supplements three kinds of factors: subjective social norms, relational factors, and social-emotional factors. Then, Fuentes-Moraleda et al. (2020) applied the sRAM model to hotel scene and verified the model by online comments. Fernandes and Oliveira (2021) and Zhong et al. (2022) empirically validated the model by online survey. In addition, de Kervenoael et al. (2020) developed the TAM model based on the key dimensions (perceived usefulness, perceived ease of use, service guarantee, personal participation, tangible assets, empathy, perceived value, and information sharing) that encourage tourists to use social robots in tourism reception and travel services. Similarly, Go et al. (2020) developed the Interactive Technology Acceptance Model (iTAM) because of introducing technical features. Based on the extended TAM, Abou-Shouk et al. (2021) also developed a model for hotels and travel agencies. The core of these models is the TAM model.

With the development of AI technology, scholars noticed the differences between intelligent technologies and non-intelligent technologies. Considering the TAM studies’ object is the acceptance of non-intelligent technologies, and service robots have human-like intelligence, Lu et al. (2019) think that TAM’s core concepts, perceived usefulness and perceived usability, are not applicable in service robot scenarios, and build a theoretical model of six dimensions (performance efficacy, intrinsic motivation, anthropomorphism, social influence, facilitating conditions, and emotion) that affect consumers’ long-term use intention of service robots, then developed and test the service robot integration willingness scale (SRIW) by online survey. Then, to further understand the relationship between different dimensions of the SRIW, Gursoy et al. (2019) proposed a three-stage AI device use acceptance framework (AIDUA) from the cognitive appraisal theory and cognitive disorder theory. Since then, Lin et al. (2019) and Roy et al. (2020) have successively verified and developed the AIDUA model in different countries. With the emergence of the COVID-19 pandemic, different research perspectives have emerged, new factors are integrated into the research of service robot acceptance. Kim et al. (2021) used experimental methods to study the impact of pandemic on service robot acceptance from the perspective of health crisis. Similarly, Kang et al. (2022) used experiments to explore how disease contagion cues shape customers’ willingness to adopt service robots.

As mentioned above, with the deepening of related research, the research on interaction between service robots and users has not only considered the related factors of perceived usability or perceived usefulness in traditional technology acceptance research, but also gradually added new elements, such as emotion, relationship, trust (van Doorn et al., 2017; Park and Tribe, 2020), and perceived threat (Kim et al., 2021), performance goal orientations (Tojib et al., 2022). A better understanding of how users interact with service robots in hotel scenarios will improve the design and future application of service robots (Tussyadiah et al., 2020). By comparing the characteristics and applicability of different theoretical models, this study holds that the six-dimension model developed by Lu et al. (2019) covers a comprehensive range of influencing factors. There are two reasons. First, this scale is aimed at the influencing factors of service robots’ acceptance, which not only includes the traditional factors of technology acceptance research, but also considers the characteristics of service robots. Moreover, it is not included in specific service scenarios, so it has a wide applicability and can be applied to the research of service robots in hotel scenarios. Second, this scale makes clear the differences of the core factors that affect consumers’ acceptance of intelligent technologies and non-intelligent technologies. The perceived usefulness and perceived ease of use in the acceptance research of non-intelligent technologies are not suitable for the acceptance intention research of intelligent technologies. Taking into account the differences between the two technologies (intelligent vs. non-intelligent), the scale screens out the factors that is suitable for the research of intelligent technologies.

In terms of analysis methods, quantitative analysis (mainly questionnaires and experiments) is the mainstream method in service robot research, although there are some preliminary qualitative studies, the research on users’ experience and feedback in real scenes is limited. Studies using qualitative data such as comments, interviews, semi-structured interviews (Seyitolu et al., 2021), or mixed methods (Chen et al., 2022; Zhong et al., 2022) can identify richer potential influencing factors from multiple perspectives, which is a supplement to quantitative studies. Compared with other qualitative research methods, the information generated by users on the Internet has some obvious characteristics, such as concentrated topics, large amount of information, relatively real information, and Easy to access publicly. Service robot is an emerging technology, although there are many usage scenarios, it is difficult to get feedback from a certain number of consumers who have used robots in specific scenarios. Therefore, online reviews have obvious advantages in studying consumers’ willingness to accept service robots.

Therefore, this study finally uses the service robot integration willingness scale to analyze user comments and explore the factors that affect hotel users’ willingness to accept service robots in China.

### Service robot integration willingness scale

According to the service robot integration willingness scale (Lu et al., 2019), the influencing factors of consumers’ willingness to accept service robots mainly depend on six dimensions: performance efficiency, intrinsic motivation, anthropomorphism, social influence, facilitating conditions, and emotion. The target technology of this questionnaire is the service robot loaded with artificial intelligence technology, so it is helpful to better understand the interaction between users and robots in hotel service scenarios.

Performance efficacy is a concept that integrates multiple concepts such as perceived usefulness, perceived ease of use, performance expectancy, and perceived effort expectancy, it reflects the degree to which robot service is superior to employee service (Lu et al., 2019). Performance efficacy not only covers the functions of the service robot, but also includes the use experience of service robots. This concept is helpful to better understand users’ evaluation of robots.

Intrinsic motivation is the pleasure that users get when interacting with technical equipment, and it has been verified in the technical use scenarios of consumers and employees (such as Brown and Venkatesh, 2005; Venkatesh et al., 2012). As a new service technology, service robot has the characteristics different from traditional technologies (such as self-service teller machines, mobile payment, etc.), and has the interactive function like that of hotel employees (Lu et al., 2019). In addition, it has a variety of features such as appearance, voice, and mobility, so that hotel service robots can bring different experiences to consumers, and consumers’ motivation will affect hotel robot deployment strategies, such as providing regular service functions to meet practical motives, providing additional interactive functions to satisfy consumers’ demand.

Anthropomorphism means that products have psychological characteristics (emotions, personalities, gestures, and other human-like appearance) and non-psychological characteristics, that is, there are similarities with human body (such as head, eyes, arms, legs, etc.) (Keeley, 2004; Schreibelmayr and Mara, 2022). There is no consistent conclusion about the role of anthropomorphism in product evaluation. For example, Kim and McGill (2011) found that anthropomorphic appearance can positively influence consumers’ attitudes and purchasing intentions, but the positive effects of anthropomorphism occur when products do not have human intelligence (Mimoun et al., 2012; Garnier and Poncin, 2013). There is also no consistent conclusion on the research of anthropomorphism of intelligent products, especially in the context of artificial intelligence and service robots. It is necessary to study anthropomorphism for understanding the interaction between consumers and technology (van Doorn et al., 2017; Murphy et al., 2019; Yu and Ngan, 2019). For smart devices, having a physical appearance like human beings may pose a threat to consumers’ human identity. People may feel uncomfortable with the idea that human beings may be losing their uniqueness, because the human similarity of robots leads to the perception of dangerous sources of human identity (Ackerman, 2016; Huang et al., 2021). There are also different views in the anthropomorphism research of service robots. For example, van Pinxteren et al. (2019) found that humanoid robots can positively influence users’ willingness to use robots through trust, while Goudey and Bonnin (2016) found that consumers’ acceptance of companion robots with partial anthropomorphic designs was higher than that of complete human appearances. On the contrary, Lu et al. (2019) and Huang et al. (2021) pointed out that anthropomorphism hindered consumers’ acceptance of service robots. Mende et al. (2019) went deeper and found that anthropomorphism would cause consumers’ compensation consumption. In view of this, it is necessary to re-examine the influence of anthropomorphism on consumers’ willingness to accept robots.

Social influence is the degree to which consumers’ social networks think they should use technologies (such as robots) in service contact. The role of social influence in the decision-making of technology adoption may be complicated and depends on the specific situation (such as customers and employees) (Venkatesh and Morris, 2000). Previous studies have shown that social influence can affect consumers’ views on technology acceptance through internalization (such as Venkatesh and Morris, 2000; Venkatesh et al., 2003). Therefore, reference groups may influence consumers’ perception of service robots and play a significant role in making decisions to support or reject this innovative technology. However, it remains to be seen whether the social influence can be extended to the field of hotel service robots. For example, Roy et al. (2020) found that the social influence will positively affect users’ willingness to use robot in Indian samples, while Lu et al. (2019) found that the social influence will not affect users’ willingness to use robots in American samples. Therefore, it is necessary to explore the role of social influence in Chinese hotel scenarios.

Facilitating conditions are those resources and assistance that promote the use of technology (Brown and Venkatesh, 2005), which includes some aspects of the technical or organizational environment, aiming at reducing the use difficulty of consumers (Venkatesh et al., 2003). Although service robots are equipped with human-like intelligence, as machines, they may not be able to fully capture the nuances of human interaction. Whether they can participate in human communication efficiently is crucial for consumers to regard service robots as human substitutes. In this process, specific conditions or resources are needed to assist the robot to perform the service smoothly. Therefore, it is essential to investigate the facilitating conditions for understanding the acceptance of service robot.

Emotion is an important topic in the study of human–robot interaction (Fong et al., 2003). Previous studies have examined the influence of emotion on technology use and found that emotion is an important antecedent and intermediary factor of technology use (Lin et al., 2019; Roy et al., 2020). In the process of interacting with products, emotions will be aroused. When the products meet consumers’ expectations, users may experience satisfaction, and when their performance exceed consumers’ expectations, this satisfaction may be further upgraded to joy (Weiss et al., 2009). Positive emotions include expectation, satisfaction, happiness, delight, joy, and surprise (Watson and Spence, 2007), while negative emotions include depression, fear, uncertainty, anxiety, and worry (Raghunathan and Pham, 1999). Therefore, when consumers interact with service robots, there may be various complicated emotional experiences. Investigating consumers’ emotional changes plays a key role in understanding consumers’ behavioral intentions.

The core of hotel hospitality service is an experience that lasts for a period, and related service runs through the whole accommodation process in hotels (such as check-in, check-out, room service, etc.). The six dimensions of the service robot integration willingness scale comprehensively consider the characteristics of consumers and service robots in various hotel scenarios and have been verified by scholars in different studies (Lin et al., 2019; Roy et al., 2020), and it is robust to a certain extent.

## Materials and methods

### Service robot Run

**Figure 1:**
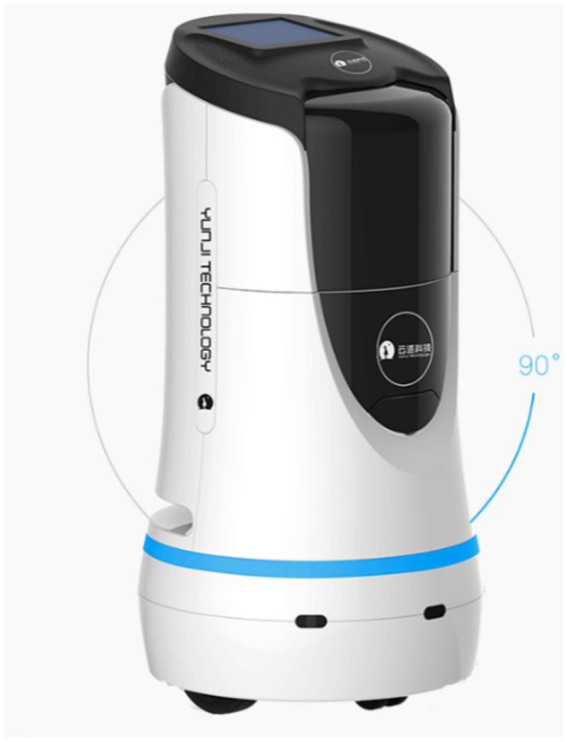
Intelligent delivery robot run. Source: https://www.yunjichina.com.cn/product_run.html. Reproduced with permission.

The application of service robots in hotel scenarios is at a relatively preliminary stage, and the existing robot types are relatively limited. In this study, Run (as shown in Figure 1), a widely used service robot, is selected as the research material in combination with the availability of service robot’ online comments and research requirements (whether it has anthropomorphic characteristics or not).

On the one hand, this service robot is an intelligent delivery robot developed by Yunji Technology, which has three functions of “running errands to deliver goods,” “welcoming and leading,” and “publicizing and broadcasting.” The website of Yunji Technology shows that this robot is used in more than 240 cities in 34 provinces, municipalities, and autonomous regions in China, covering more than 20 countries and regions such as Europe, America, South Korea, Japan, and Thailand. This robot has different application functions, and numerous hotels use it, so it can be considered that there are numerous online reviews. On the other hand, according to the robot classification standards of Tung and Law (2017) and Čaić et al. (2019), Run has anthropomorphic expression and voice functions, and its overall appearance is close to cartoon and functional robots. Therefore, Run is the object that meets the research requirements.

### Data processing methods

This study used the content analysis method, referring to Fuentes-Moraleda et al. (2020), and the specific method was divided into five stages. The first stage was divided into four steps. In the first step, the official website of Yunji Technology shows the names and distribution of typical hotels that use service robots in different areas of China. Using this hotel list, online comments of each hotel were collected on Qurna (Qunar.com). Qunar is one of the largest Chinese online travel agencies (OTA) platforms in the world and allows anyone access to its reviews, thus providing us with a rich source of data for analysis. In the past, service hospitality studies have used the comments of online booking platforms for human–robot interaction analysis. For example, Çakar and Aykol (2021) used the comments of TripAdvisor for analysis, Tung and Au (2018) collected the user comments of TripAdvisor, Agoda, Yelp, and Booking.com, while Park et al. (2021) collected reviews from Chinese customer review platform Dazhongdianping. The second step was to screen out the hotels that mentioned “robot” or “robot waiter” in online reviews. The third step was to further verify whether the robot information mentioned in the comment information is related to service robots, such as “the service is not enthusiastic, generally speaking, it is similar to the robot, and there is no smile.” Once it is confirmed that robots are mentioned in hotel reviews, we searched for robot nicknames mentioned in the reviews (such as Xiaomei, Dabai, Coral Sister, Xiaorui, etc., not all hotel robot reviews mention nicknames, and a total of 26 hotel reviews mention robot nicknames) and searched the reviews again in combination with robot nicknames. During the fourth step, through repeated screening in the first three steps, we selected hotels with a total number of comments (robots or robot nicknames mentioned in the comments) more than five as samples, this criterion was informed by Fuentes-Moraleda et al. (2020), and other comments were excluded. Only Chinese comments are considered when selecting comments.

The list of some cooperative customers of Yunji Technology official website showed 95 hotels that use Run, there were 92 hotels on Qunar before September 6, 2021, among which five hotels cannot be collected. Therefore, the actual number of hotels collected is eighty-seven, distributed in 29 provinces, municipalities, and autonomous regions in China, with a total of 234,067 comments, including the following fields: username, number of comments, comments, and visited destination. The comments are extracted from Qunar through Houyi collector (a data crawling software).

Through the screening of the above four steps, the final sample (Table 1) contained 4,107 comments from 68 hotels, which were published before September 6, 2021. The samples of hotels are homogeneous. Most hotels are 4-star or 5-star, and each hotel uses at least one service robot. Hotels are distributed in 24 provinces, municipalities, or autonomous regions in China.

**Table 1:** Research fact sheet.

Hotel selection	Object	To select hotels in China that had at least one Run service robot implemented
	Hotel characteristics	Method of selection: according to the official website of Run, find the typical hotels that use this robot
		Number of hotels: 68
		Hotel type: three stars 1.5%, four stars 25%; five stars 73.5%
	Robot function	Guiding service and delivery service
	Platforms	Qunar (for hotel selection and reviews)
		Yunji (for robot viewing and classification)
Reviews	Object	Reviews included in the study mentioned a robot, robots, or robot nickname
	Number	4,107
	Date	The comments before September 6, 2021
	Analysis method	Content analysis. Sentiment analysis. Frequent words analysis. Classification of content was performed manually

The second stage included importing data into NVivo v.12 software for content analysis. The data of each hotel was a separate Excel table, which only contained valid comments. All hotel tables were imported before being analyzed. Content analysis has been used in previous hotel studies to verify the applicability of the SRAM model (Fuentes-Moraleda et al., 2020), explore users’ robot experience (Tung and Au, 2018), and measure users’ evaluation of robots (Yu, 2020). Therefore, content analysis is a suitable research method for comment information analysis.

When all comments containing “robots” or “robot nicknames” were compiled into the software, the third stage used NVivo v.12 to identify the 500 words with the highest frequency in comments. Among the initial 500 words, the words associated with the term “robot” or “robot nickname” were manually recognized. The screening criteria of high-frequency words were as follows, (a) removing irrelevant scene description words (such as “hotel” and “this”); (b) merging the high-frequency words with similar meanings (not only similar literal meanings, such as “children” and “child,” but also consider whether the words are describing similar scenes) according to the clustering of the initial dendrogram, in this step, we needed to find the specific content of comments through software; (c) according to the number of clusters initially set by the software (the initial clustering of the software shows 100 high-frequency words, 10 subclasses, and up to 20 subclasses can be set), adjusting the clustering results based on the merged high-frequency words; and (d) repeating the operation of the first two steps, and further screening the high-frequency words directly related to robots by comprehending the specific context of the comment each high-frequency word located, which reduces the initial number of high-frequency words to 100.

The fourth stage was content analysis, which classifies 100 recognized words related to “robot” or “robot nickname” manually. To ensure the anonymity of hotel information and simplify the screening of high-frequency words, we had replaced each “robot nickname” with “robot.” The classification of high-frequency words was based on the six dimensions of service robot integration willingness scale.

The fifth stage included dimension analysis and sentiment analysis of the whole sample. NVivo constructed a hierarchical cluster diagram (tree diagram). In the tree diagram of cluster analysis, highly related words are grouped into a cluster hierarchy (Chan et al., 2017). According to Ceglia et al. (2017), data clustering based on the similarity of coded content is usually suitable for qualitative research.

Sentiment analysis was also conducted using NVivo v.12. Sentiment analysis is a method to examine the sentiment of samples, to explore different views presented in the information (Fuentes-Moraleda et al., 2020). Based on the content, NVivo software classifies sentiments into four categories: (a) very negative, (b) moderately negative, (c) moderately positive and (d) very positive.

## Results

### Findings by sentiment analysis

A sentiment analysis was conducted on all user comments, to identify the sentiment of consumers interacting with robots in hotels. The analysis was performed by coding phrases. The results showed that 7.73% were very negative, 7.01% were relatively negative, 42.38% were relatively positive, and 42.87% were very positive. The results showed that the overall evaluation of service robots by users was positive (85.25%), which verified the previous findings (Seyitolu et al., 2021; Ayyildiz et al., 2022; Filieri et al., 2022; Zhong et al., 2022). Then, the sentiment analysis of sub-dimensions was conducted, and the analysis results were shown below.

### Findings by dimensional analysis

As the high-frequency words identified in the third part were not related to the concept of social influence, it contradicts the findings of Gursoy et al. (2019), Lu et al. (2019), Lutz and Tamò-Larrieux (2021). One of the possible reasons is that most users do not know that hotels provide robot services before they check into hotels, the human–robot experience is out of expectation, so the social influence does not work. The other reason is that all reviews are from mid-range and high-range hotels in this study, and this is consistent with the finding of Lin et al. (2019), social influence has limited impact on users’ willingness to use robots in full-service hotel.

Subsequently, this study divided 100 high-frequency words into five categories, and the comments of high-frequency words accounted for 89.29% of the total number of comments, which was representative. The most frequently mentioned by users is performance efficiency (55.39%), followed by intrinsic motivation (40.08%), then anthropomorphism (29.36%) and emotion (28.27%), and finally facilitating conditions (23.17%). The grouping of words related to each dimension mentioned by users in comments is shown in the following tree diagram, which analyzes the correlation between the selected words. The following figures showed the results of five clusters in turn, which is automatically generated by word meaning clustering (this result is generated by Pearson correlation coefficient.) function in NVivo 12.

#### Intrinsic motivation

**Figure 2:**
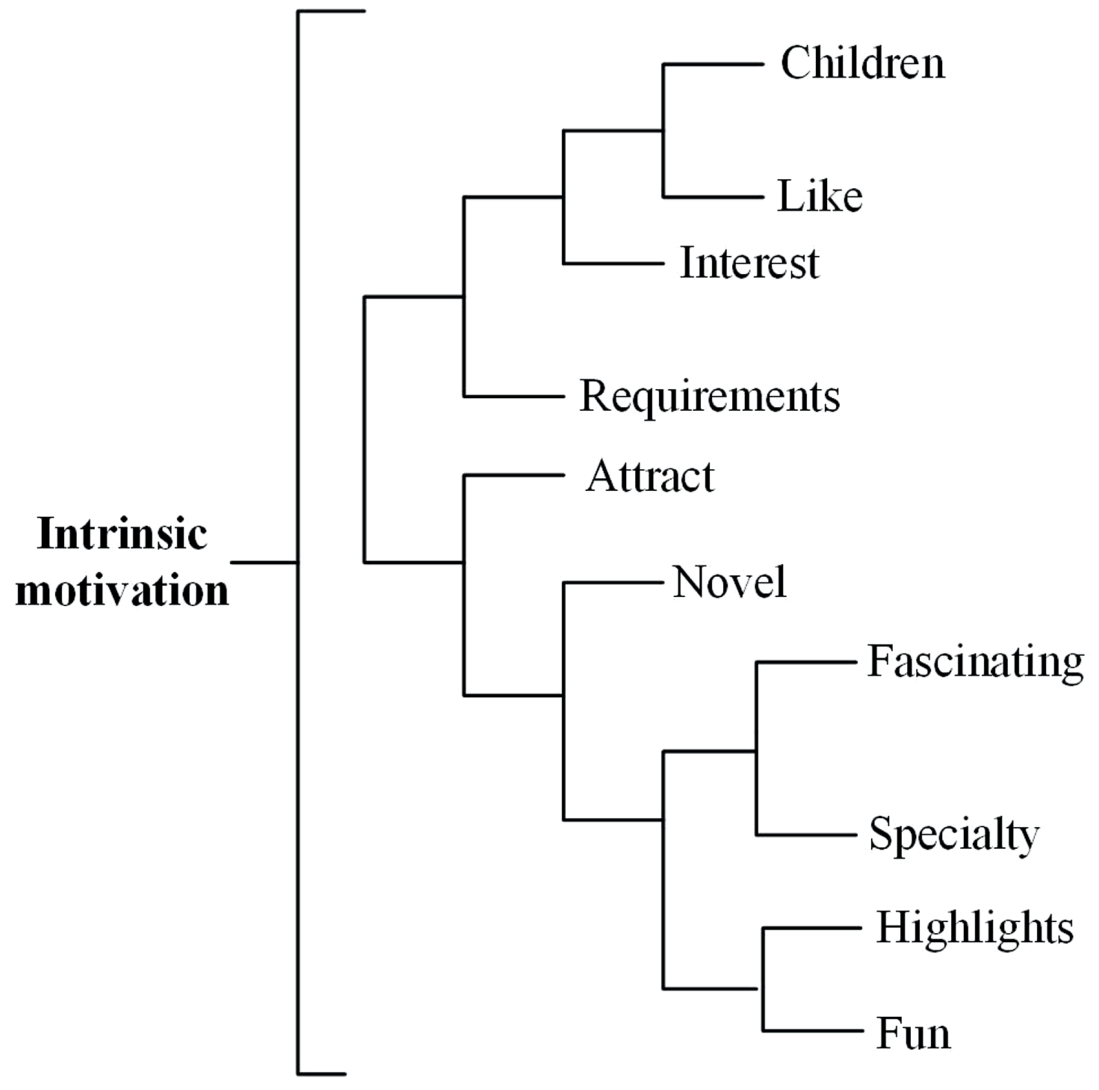
Intrinsic motivation dimension dendrogram.

According to Gursoy et al. (2019), the definition of hedonic motivation is “perceived pleasure or pleasure that individuals expect to get by using AI devices in service delivery,” intrinsic motivation is mainly reflected as “hedonic motivation” in high-frequency words (Figure 2). Different from the findings of Fuentes-Moraleda et al. (2020), comments not only reflect the interaction between children and robots, but also include the interactive contact between adults and robots. Studies have shown that children are particularly sensitive to the interaction with robots. For example, Pinillos et al. (2016) found that children are closer to robots than adults and tend to interact with robots more frequently.

One of the highlights of this hotel is the super cute robot. Even adults like me love this robot very much, so the hotel is very suitable for families with children. (Fujian province, anonymous)

To experience the robot service, I asked the reception to give me water and slippers, which was quite fun. (Beijing, anonymous)

The comments also show that children’s positive attitude toward robots influences parents’ attitudes, which is similar to the study of Tung and Au (2018). Robot service strengthens the relationship between parents and children and increases the content and value of hotel experience; for the interaction between children and robots, parents are even willing to accept a certain degree of service failure (such as slow check-in speed).

Children like hotel robots best, and always call customer service for delivery. (Beijing, anonymous)

In-store robot delivery is quite novel, and children like it, so they tried it several times specially (this review is just to let the robot mother give her candy). Suggestion: Robots can further optimize the content of human–robot interaction. (Beijing, anonymous)

The results of sentiment analysis (Table 2) show that 86.98% of users have a positive evaluation of robots in the dimension of intrinsic motivation, which is similar to the findings of Lu et al. (2019) and Roy et al. (2020), and hedonic motivation has a positive impact on service robots’ willingness to use.

**Table 2:** Sentiment analysis of intrinsic motivation dimension.

N valid cases = 1,646	Very negative (%)	Moderately negative (%)	Moderately positive (%)	Very positive (%)	Total (%)
	6.79	6.23	40.19	46.79	100

In terms of motivation, very positive and moderately positive emotions dominate reviews (Table 2). According to the cluster analysis obtained in the tree diagram, it can be observed that the aspects of motivation that cause positive emotions are “children,” “fun,” “like,” “attract” and “highlights.”

There is also a robot in the hotel that can deliver the needed items for guests. Children are very excited about this. (Anhui province, anonymous)

The negative emotion in motivation is mainly related to the failure of robot service to meet users’ demands, which is mainly reflected in the word “requirements.”

It's not convenient for guests to come down and get the takeout yourself. Why not let the robot in the lobby deliver the takeout upstairs. When staying in other hotels in other cities, some of the hotels use robots deliver takeaways. (Jiangsu province, anonymous)

#### Performance efficacy

**Figure 3:**
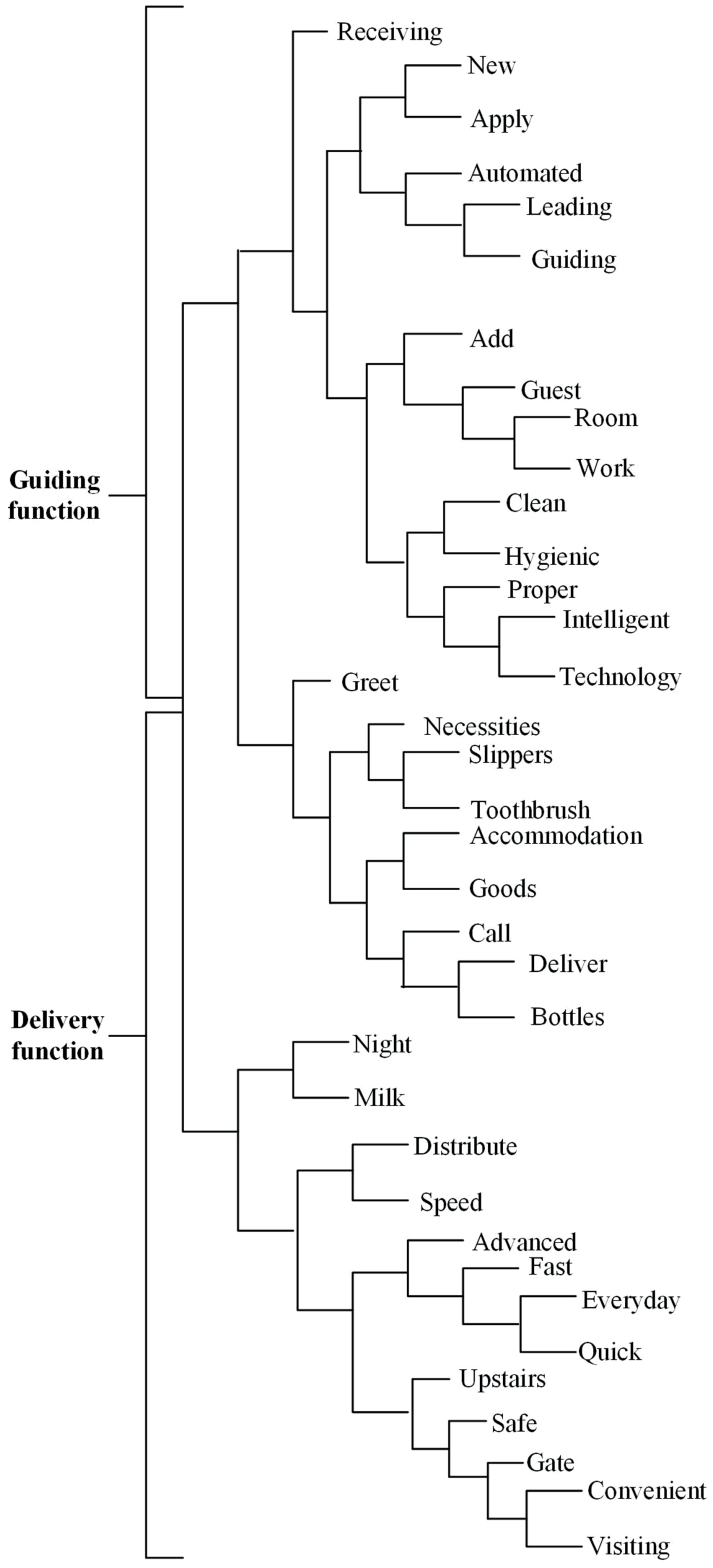
Performance efficacy dimension dendrogram.

Performance efficacy can be divided into two categories: “guiding function” and “delivery function” (Figure 3). The number of words used by users to describe the two categories are not equal, and there are more words related to delivery services.

The first category is guiding function. Although the number of high-frequency words is relatively small, it is necessary to divide the guidance function into independent categories, which is to emphasize the importance that users attach to the guidance function of robots. This has been verified in previous studies, such as Rodriguez-Lizundia et al. (2015), Pinillos et al. (2016) and Fuentes-Moraleda et al. (2020) have similar findings. The guiding function of the robot is discussed in detail in the comments, and the related comments mentioned “room,” “guide” and “welcome,” etc.

As soon as you enter the hotel door, a robot greets guests, which is very personalized. (Guangxi province, anonymous)

The second category is the delivery function. In this category, the user comments mention the specific contents of the robot’s participation in room service, such as food delivery/take-out, night service, free delivery, etc. In addition, the robot could call the user in advance before the delivery items arrive. These functions are reflected in the improvement of service experience brought by robot service. Comments mention that robots can cooperate with service personnel to provide delivery and meal services to users all day.

There is a food delivery robot, and the concierge delivers takeout to the room 24 hours a day. It is awesome! (Beijing, anonymous)

There are robots in the hotel to deliver water, slippers, and necessities, all of which are free. This is very good, and children like it very much. (Jiangsu province, anonymous)

Compared with previous studies, the customers’ description of the robot’s function in this study is more detailed. The “safe” and “intelligent” mentioned in the comments corroborate the findings of Tussyadiah and Park (2018), namely, perceived intelligence and perceived security affect users’ willingness to accept robots in room service. In addition, similar to the findings of Jia et al. (2021), safety positively affects users’ satisfaction with robots, which will affect users’ evaluation of hotels in turn.

Overall, the hotel is very safe. Take-out is delivered by robots, and we will check in next time. (Guangdong province, anonymous)

According to the sentiment analysis (Table 3), the overall evaluation of the delivery function and the guiding function of the performance efficacy dimension by users is relatively positive. Among them, 80.6% of the users have a positive evaluation of the robot in the delivery function and 81.43% of the users have a positive evaluation of the robot in the guiding function. In contrast to the findings of Lu et al. (2019), performance efficacy has a positive impact on the willingness to use service robots.

**Table 3:** Sentiment analysis of performance efficiency dimension.

N valid cases = 2,275	Cluster	Very negative (%)	Moderately negative (%)	Moderately positive (%)	Very positive (%)	Total (%)
	Guiding function	11.01	7.56	40.14	41.29	100
	Deliver function	11.22	8.18	39.14	41.46	100

In the guiding function, positive emotions mainly include words such as “leading,” “room,” “welcoming” and “operation.” These words are all related to the service of robots guiding customers to rooms.

Children are very happy when they enter the room with robots to guide them. (Beijing, anonymous)

Negative emotions also appear in the words “leading” and “room” of the guiding function, and related comments depict the specific situation when the robot service failed.

The thing I want to jibe most is the artificial "mentally retarded" robot, a robot was set up at the front desk to guide us to the room. The hotel is surprisingly large and built on the hillside. The reception is on the fourth floor, and we live on the second floor. The robot walked from the lobby to the room for nearly half an hour, and we followed it slowly with our luggage. The robot entered the elevator in the middle, and our luggage couldn't be pulled in!!! (Hainan province, anonymous)

In the delivery function, positive emotions mainly include words such as “intelligent,” “delivery,” “gate” and “convenient.” These words are all related to the practicality of robot delivery to room service.

When I arrived at the room, I accidentally found that there was a micro-mall in the hotel. I tried to order instant noodles, but I didn't know if it could be delivered at this late hour. After about 5 minutes, I heard something at the door. It was so interesting to see a robot waiter actually delivered the goods to my door. (Anhui province, anonymous)

However, the delivery service has also caused negative emotions, and the corresponding vocabulary was “night,” such as the failure of the delivery service.

The next night, I ordered the robot service, but after waiting for a long time, the phone rang in my room, however, after opening the door, there was no robot, it also tossed twice. Then I waited for a long time and called the front desk, the staff told me to wait a little longer. As a result, it took me a lot of time before the robot arrived at my room, which added trouble and affected my rest. (Jiangsu province, anonymous)

#### Emotion

**Figure 4:**
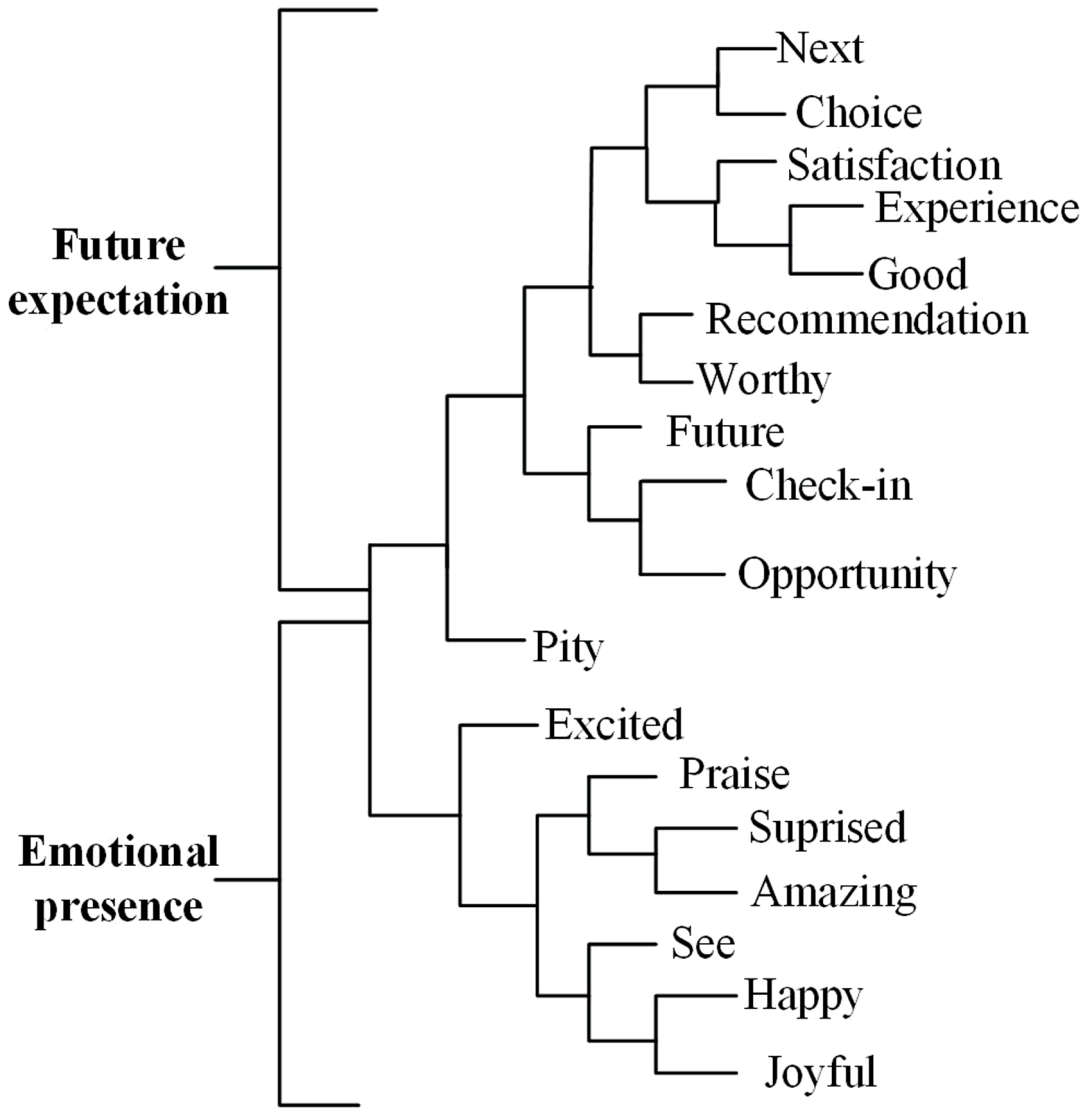
Emotion dimension dendrogram.

The two branches of emotion are “future expectation” and “emotional presence” (Figure 4). Among them, “Future expectation” contains all users’ cognitive judgments related to emotional factors, which is based on the expectation of robot services. The related words are “next,” “recommendation,” etc.

Every day, friends came to my room and said that this hotel hides its light under a bushel, and there were robots delivering takeout, very recommended, and I will come again next time. (Anhui province, anonymous)

The second category is “emotional presence,” which refers to the emotions generated by users when they are received and welcomed by robots in hotels, using robot services or seeing robots. This finding is consistent with the findings of previous studies (Tung and Au, 2018; Filieri et al., 2022). These comments contain rich emotional descriptions after human–robot interaction, covering positive and negative emotions, among which positive emotions are the primary, such as “happy,” “excited” and “pity.”

I called the hotel to deliver children's slippers, but the robot delivered them to the door, and the children were extremely happy. (Fujian province, anonymous)

According to the sentiment analysis (Table 4), users’ overall evaluation of emotional dimension’s future expectation and emotional presence is relatively positive, which is consistent with the findings of Gursoy et al. (2019), Lu et al. (2019), and Roy et al. (2020), and emotion has a positive impact on consumers’ willingness to use service robots. In the future expectation, 77.95% of users have a positive evaluation of robots, and in the emotional presentation, 79.13% of users have a positive evaluation of robots.

**Table 4:** Sentiment analysis of emotional dimension.

N valid cases = 1,161	Cluster	Very negative (%)	Moderately negative (%)	Moderately positive (%)	Very positive (%)	Total (%)
	Future expectation	12.03	10.03	38.21	39.74	100
	Emotional presence	9.91	10.96	39.14	39.99	100

In the future expectation, positive emotions mainly include words such as “satisfaction,” “recommendation,” “next,” and “experience.” These words are all related to the service function of robots. Among them, “satisfaction” has been confirmed in the previous study (Li, et al., 2010).

There are robots that deliver takeout and disposable appliances to the room, which is very distinctive. Children like it very much, and they will check in next time! (Anhui province, anonymous)

However, the future expectation also contains negative emotions, such as “future” and “choice.” These words are related to the unpleasant experience of robots.

Robot delivery is not very easy to use either. When it arrived at the door, there was no doorbell ringing, which take me more than 40 minutes to get a takeout from the lobby to my room. During this period, I called the front desk to urge it many times, which was awfully bad. I will not consider this hotel in the future. (Guangdong province, anonymous)

In emotional presence, positive emotions contain words such as “happy,” “joyful,” and “excited.” These words are related to the user’s human–robot interaction experience.

Robots are particularly attractive to children. They have been called for several times, and the children are incredibly happy. (Beijing, anonymous)

Negative emotions appear in the words “pity” and “see,” but not all the contents described are unsatisfactory service experiences.

I like the little robot that can deliver water best. It's especially cute. It's really fun while delivering water. Unfortunately, I forgot to take a picture of it, which is a pity. (Jilin province, anonymous)

#### Anthropomorphism

**Figure 5:**
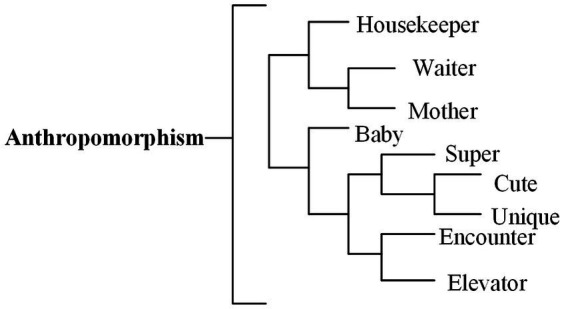
Anthropomorphism dimension dendrogram.

Although there is no obvious distinction in the vocabulary of dendrogram, anthropomorphism is reflected in the “shape” and “function” of the robot (Figure 5). The anthropomorphism of the robot’s shape is manifested in the design of the robot itself, with human-like eyes and expressions. de Kervenoael et al. (2020) pointed out that visibility can positively influence consumers’ willingness to use robots. Similar to previous research findings (Li et al., 2010; Tussyadiah and Park, 2018; Lin, et al., 2019), the description in the comments proves that the appearance features will bring users a more unique experience, and then users have a more positive evaluation of robots.

The adorable robot can lead the way, and it can also talk to you, which is especially cute. (Fujian province, anonymous)

On the day of check-out, I saw a cute and busy robot leading the guests to check out. Because there were many people, it was anxious at the elevator door. (Beijing, anonymous)

The anthropomorphic function of the service robot is reflected in the fact that the robot can take the place of employees to go upstairs and downstairs, avoid roadblocks and deliver goods independently through wireless network. At the same time, the robot has voice function, and the anthropomorphic voice is used to realize the interaction between users and robots during the interaction process. Although it is not completely two-way communication, it can provide users with more convenient room service.

There is a lovely food delivery robot in the hotel, and I had a good time with him. Before entering the elevator, the robot would say, "I'm so nervous!" Ridiculously cute! (Beijing, anonymous)

The anthropomorphism of robots has obvious positive effects on users’ use of robots, both adults and children have positive comments on the anthropomorphism of robots. This is inconsistent with the conclusion drawn by Lu et al. (2019) in the hotel scenarios.

According to the sentiment analysis (Table 5), 85.62% of users have a positive evaluation of robots in the anthropomorphic dimension, which is consistent with the findings of Gursoy et al. (2019) and Roy et al. (2020) and contrary to the results of Lu et al. (2019).

**Table 5:** Sentiment analysis of anthropomorphic dimensions.

N valid cases = 1,209	Very negative (%)	Moderately negative (%)	Moderately positive (%)	Very positive (%)	Total (%)
	8.7	5.68	37.51	48.11	100

In the anthropomorphism dimension, positive emotions include words such as “cute,” “waiter,” “housekeeper” and “baby.” These words are closely related to the shape and function of robots.

On the first night, I called the guest service centre and asked for slippers for my children. After a while, I got a call saying "Hello, I'm a robot. I've arrived at the door of your room. Please open the door." The child excitedly opened the door and took out the slippers according to the voice prompt of the robot. When the robot left, it said, "Please give me a favourable comment, and my mother will give me candy to eat." Ha-ha, what a humorous and cute robot! (Shanghai, anonymous)

However, anthropomorphism also contains negative emotions, such as “elevator” and “encounter.” These words are related to the terrible experience of robots.

The little robot broke down occasionally. Yesterday, it dominated the elevator on the first floor and prevented the guests from entering. As a result, other elevators didn't stop at the first floor. Finally, the guests had no choice but to force their way in. (Jiangxi province, anonymous)

#### Facilitating conditions

**Figure 6:**
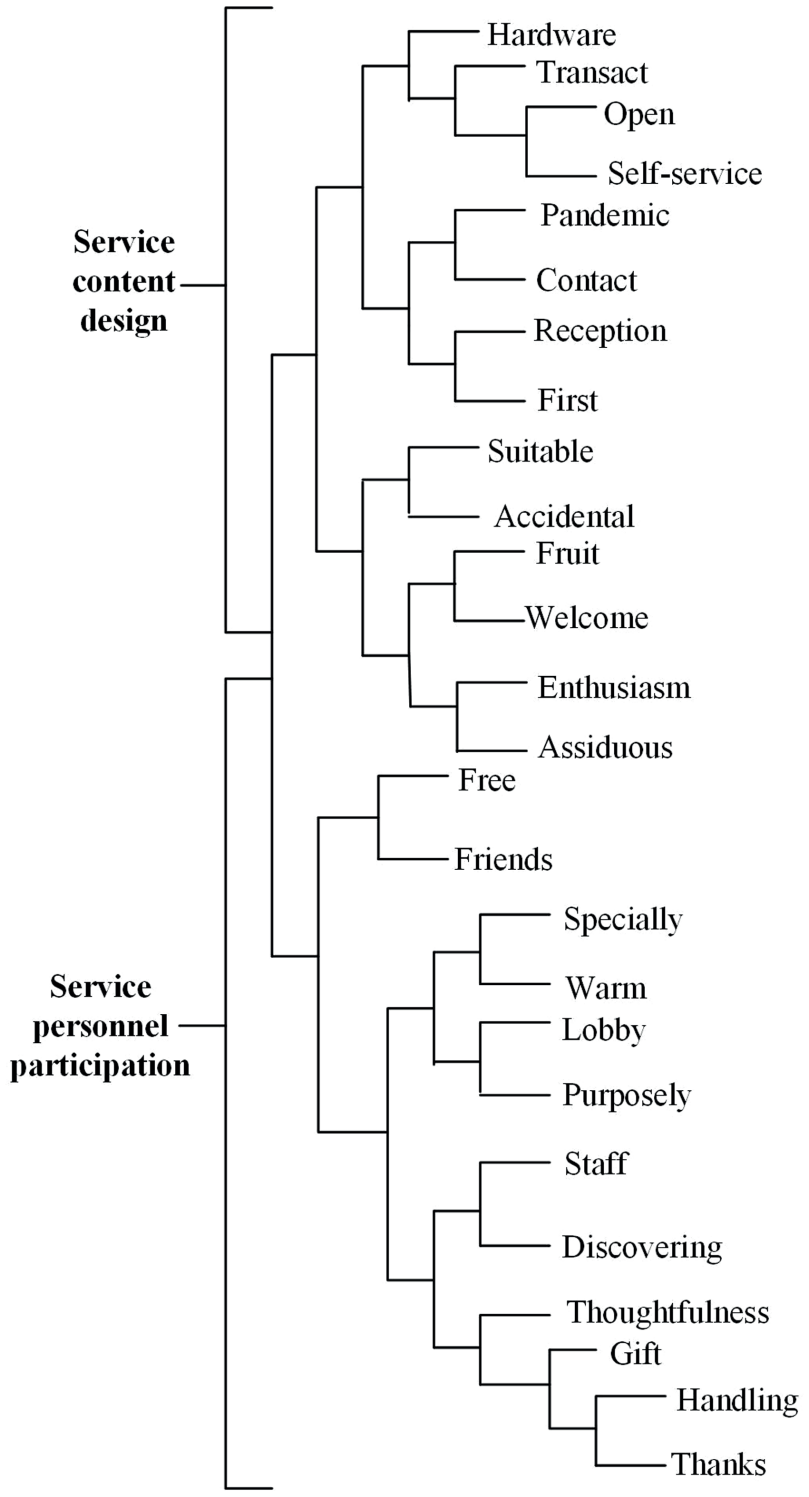
Facilitating conditions dimension dendrogram.

The two categories of facilitating conditions are “service content design” and “service personnel participation” (Figure 6). In the “service content design,” high-frequency words are reflected in how hotels incorporate robots into the whole service process, connect robots with the original services, add various service links, and bring new experiences to users, instead of completely replacing employees.

If you want mineral water, you can call the service centre, and the robot will deliver the water. It's very advanced, and children will like it. (Beijing, anonymous)

In addition, service content design is a crucial factor for customers to examine service quality. In the comments, it is shown as a small gift in the welcoming process, or the first contact between the user and the robot.

There is a robot in the hotel, although it can't chat with each other, but the robot will give welcome fruit to the room, which is a personalized service. (Guangxi province, anonymous)

The first time I stayed in this hotel, the service was very good, and the welcome fruit presented by robots was an enjoyable experience. (Guangxi province, anonymous)

In the “service staff participation,” hotel staffs’ participation in service is considered as an important part of robot service. In this process, service staff make full use of robots, and the active participation of service staff improves users’ evaluation of hotels. Previous studies have been verified, such as Choi et al. (2020) found that in hotel service environment, users’ evaluation of robot service with employees’ participation is higher than that provided by service robot alone in terms of interaction quality.

The customer relations director was very enthusiastic. Seeing that the children were interested in hotel robots, he asked the robots to send us some small items such as toothbrushes. It was highly intelligent, and the children liked it very much. (Beijing, anonymous)

According to the sentiment analysis, users have a positive overall evaluation of service content design and service personnel participation in the facilitating conditions dimension (Table 6), and the facilitating conditions have a positive impact on the consumers’ willingness to use service robots, which is consistent with the findings of Lu et al. (2019) and Roy et al. (2020). Specifically, in the service content design, 74.52% users have a positive evaluation of robots; in the service staff participation, 79.68% users have a positive evaluation of robots.

**Table 6:** Sentiment analysis of facilitating conditions dimension.

N valid cases =949	Cluster	Very negative (%)	Moderately negative (%)	Moderately positive (%)	Very positive (%)	Total (%)
	Service content design	15.08	10.04	43.49	31.03	100
	Service staff participation	12.05	8.26	40.27	39.41	100

In the service content design, the vocabulary of positive emotions includes “pandemic,” “suitable,” “contact” and “hardware.” These words are all related to the service provided by robots. Especially after the outbreak of COVID-19, due to the need of epidemic prevention, to reduce the risk of infection, and avoid close contact between people, the service robot that replaces employees has more practical value, rather than just business strategies or gimmicks. This finding is in line with those previous studies in which the pandemic was identified as the positive influencing factor for robot acceptance (Ab Rahman et al., 2022) and customer-robot engagement (Wu et al., 2021).

There are robot waiters in the hotel to prevent and control the epidemic and reduce contact, and the service is in place! (Guangdong province, anonymous)

However, the reviews of service content design also contain negative emotions, such as “reception” and “self-service.” Confirming the views of Filieri et al. (2022), these words are related to the difficulties users encountered in using robots.

There is a service robot in the lobby, which can only be used if the reception clerk inputs a password. At that time, when booking a room, I saw that the robot was leading other guests to their room, however, when it was my turn, I had to go to the room on my own. Later, when I went downstairs to take my children to experience the robot, the clerk saw I was studying the use of the robot, but he did not enthusiastically teach us how to use and input a password for our use. This is the only deficiency. (Shanxi province, anonymous)

In the service staff participation, positive emotions include words such as “enthusiasm,” “thoughtfulness,” “warm,” “caring,” “staff” and “thanks.” These words are all related to the service content provided by service personnel.

There are robot waiters in the hotel to prevent and control the epidemic and reduce contact, and the service is very suitable! (Guangdong province, anonymous)

However, negative emotions such as “handling” and “discovering” are also involved in the service personnel participation. These words are related to the unreasonable use of robots by service personnel, in line with the previous study (Honig and Oron-Gilad, 2018).

The hotel's fast service is useless, the speed of answering calls is fast, but the speed of dealing with problems is slow, the delivery of goods depends entirely on robots, and the response time is more than half an hour, so it needs to be urged many times. I hope it will be improved later. (Guangdong province, anonymous)

## Conclusions and implications

### Conclusion

From the review analysis, it can be concluded that the overall evaluation of Chinese mid-range and high-range hotel users is positive for the guidance and delivery services provided by robots. The most frequently mentioned comments by users are performance efficacy and intrinsic motivation, followed by anthropomorphism and emotion, and then facilitating conditions. All five dimensions positively influence users’ evaluation of service robots; however, the comments do not reflect the influence of social influence dimension on human–robot interaction evaluation. With these results, the research questions can be answered as summarized in Table 7.

**Table 7:** Research questions and answers.

Research question	Answer
RQ1. Should service robots be introduced into mid-range and high-range hotels in China?	Yes. The overall evaluation of service robots in comments posted by 4,107 users from 68 hotels of Qunar is positive.
RQ2. What are the most frequently mentioned dimensions (factors) that affect consumers’ willingness to accept service robots in Chinese mid-range and high-range hotels?	The most frequently mentioned dimension by users is performance efficacy (guiding function and delivery function), followed by intrinsic motivation(hedonic motivation), anthropomorphism (moderate), and emotion (future expectation and emotional presence), finally, the facilitating conditions (service content design and service personnel participation), the five dimensions have positive impact on users’ evaluation of service robots

In the intrinsic motivation dimension, adults and children mostly use robots out of interest. Most users are in actual contact with robots for the first time, and they do not know much about the functions of robots. This phenomenon can be reflected in the interactive evaluation description between children and robots. Researchers and practitioners agree that it is a trend for hotel industry to introduce robot services (Murphy et al., 2017; Bowen and Morosan, 2018; Yu, 2020). Therefore, the deployment of service robot can form a competitive advantage in the industry competition for hotel enterprises. In addition, hotels should fully display the characteristics of robots, further improve the exposure of robots, and provide more opportunities for interaction between users and robots.

In the performance efficacy dimension, users pay the highest attention to the function of the robot. Although the function of this robot is limited, a few users find that the robot cannot conduct two-way voice communication, the storage capacity is small, but most users still give positive evaluation to the existing robot function. Robot service is an important supplement to human service (such as round-the-clock room service). Making full use of various functions of robot and closely combining it with hotel service is an important way to improve user evaluation. With the development of robot technology, it is also the direction of hotel enterprises to optimize and upgrade the application of existing robot technology and provide more novel and appropriate services for users.

Future expectation and emotional presence in emotion dimension reflect the high evaluation of robots by users. In the future expectation, it can be found that users’ positive evaluation of robots will affect their subsequent intentions to stay or recommend robots. This is similar to the findings of Jia et al. (2021). Therefore, for hotels, satisfactory robot service will not only make users give positive comments to robots, but also bring positive comments to hotels and introduce potential hotel customers.

The anthropomorphism dimension is not described in detail in the reviews, but as far as the robot’s appearance is concerned, the users’ evaluation of the partially anthropomorphic robot is more positive, which is consistent with the findings of Goudey and Bonnin (2016), Jia et al. (2021), as well as the description of the uncanny valley theory (Mori et al., 2012). The proper voice system function increases the anthropomorphism of the robot and does not disgust the user. From the analysis results, anthropomorphism positively affects users’ evaluation of service robots, which is contrary to the conclusion drawn by Lu et al. (2019) in the hotel scenarios.

Service content design in facilitating conditions dimension is the first step of robot deployment, and the robot’s location arrangement and service category arrangement are the prerequisite for the smooth realization of human–robot interaction. The core of hotel service is still the service provided by employees. A better combination of employees and robot services can not only reduce the cost of the hotel, free employees from redundant services and deal with more complex service contents (Kuo et al., 2017; Wang et al., 2022), but also provide more satisfactory services for customers.

In the hotel scene, how to enhance the role of social influence is a topic that needs to be explored. A considerable number of users in the review are new to hotel service robots, and their knowledge of robots is preliminary. Consumers did not know the existence of robots until they arrived at the hotels, which is related to the weak promotion of the hotels. At present, the setting of hotel reservation page fails to reflect the hotel differentiation. It is imperative to cultivate robot culture. It is not only related to the publicity of hotels, but also closely related to social reality. The pressure of prevention and control in the post-epidemic era has a promoting effect on the use and promotion of robot services. Therefore, hotel enterprises should seize the opportunity to introduce robot services in a timely manner, which will reflect their responsibility and advanced business concept.

### Theoretical implication

The theoretical contribution of this study is threefold. First, this study is an extension of the application boundary of the SRIW scale. The conclusion of the SRIW scale is still inconclusive. Using consumers’ practical use experience, this paper verifies that the five concepts of the scale affect hotel users’ acceptance of robots in China and expands the application boundary of the SRIW scale. The findings of this study are different from those of previous studies. For example, Lu et al. (2019) identified social influence and intrinsic motivation in the scale, which could affect consumers’ acceptance of service robot in American hotel scenes, while this study identified five influencing factors, but the effect of social influence on consumers’ acceptance intention has not been found. The possible reasons are that the robots (function and shape) used exist difference, and consumers have different individual characteristics (such as culture and class). Second, this study has contributed to the anthropomorphism theory. By selecting a specific robot, this study found that the moderate degree of anthropomorphism is acceptable to consumers, which is consistent with previous studies (Goudey and Bonnin, 2016; Jia et al., 2021) on the uncanny valley theory and anthropomorphism theory. Third, this study advances service robot research in mid-range and high-range hotels. In line with previous research about online reviews (Fuentes-Moraleda et al., 2020), this study shows that it is feasible to introduce robot service into mid-range and high-range hotels in China, and the deployment of robots promotes the positive evaluation and recommendation of consumers. In addition, this study also identifies key factors of human–robot interaction, which expands the research scope of service robots.

### Practical implications

Hotel managers can use service robots to improve customers’ hotel experience. Although previous studies have suggested that consumers may consider robots as a marketing gimmick (Tung and Au, 2018), hotel managers held negative attitude toward the benefit of robot implementation (Seyitolu et al., 2021; Zhong et al., 2022), the proportion of positive evaluation is remarkably high in this research, for users who travel with children, the existence of robots is one of the crucial reasons affect family’s choice. Hotels able to invest in this technology or have already used robots should increase the promotion of robot services, adopt advertising marketing strategies, or multi-channel user-generated content for publicity. According to Chan and Tung (2019), consumers have a high evaluation of robot services in budget and mid-range hotels, but the evaluation of luxury hotels is not affected by robot services. However, from the results of this study, it is found that the overall evaluation of users is positive in mid-range and high-range hotels which deploy service robots. Therefore, providing robot services to users is probably an effective marketing strategy for mid-range and high-range hotels. Hotels can refer to the robot features and functions in this study to choose the appropriate robot style.

In addition, hotel managers should change the way they apply robots. Although robots are currently used in specific fields, their functions are limited. For example, “it cannot chat with each other” is mentioned in the comments. Hotels can explore the possibility of robots and employees performing complementary functions together, to avoid the failure of human–robot cooperation services mentioned in the comments. Designing hotel application scenarios that are more suitable for robot services is not simply a substitute for employee services, but a supplement to employee services. In order to supplement, personalize, and improve human–robot interaction and customer service, the implementation of robots in hotels should be managed from the perspective of relationship. This kind of interaction should be supported and executed by human employees, not just robots, to create a unique customer experience.

### Limitations and future research

This study contains some limitations. The first limitation is related to data. This study only collected data from Qunar, only Chinese comments are considered, and comments from other languages are not considered. In addition, nearly 86% of users did not disclose their personal information, which made the comparative analysis of user types infeasible. The second limitation is related to analysis software. When NVivo software processes the text, there exists deviation in the analysis of Chinese sentences, which cannot accurately identify consumers’ evaluation of robots in specific contexts, specifically, when the comments are just short sentences, like what Fuentes-Moraleda et al. (2020) found in English comments. In addition, NVivo’s sentiment analysis results are not completely consistent with the real sentiment expressed in the comments.

There are serval directions for future research. First, future research can collect panel data and analyze the evolution of the importance of each dimension contained in user comments over time, to understand the problems that users may encounter in divergent stages of accommodation and can also focus on how culture influences consumers’ willingness to accept robots by collecting consumers’ individual data. Second, future research can compare the differences of reviews of different hotel types in various dimensions, and further explore the complex relationship between different dimensions and the influencing mechanism of different dimensions on user evaluation. Third, as in previous studies (Li and Peng, 2022), future research can continue to explore whether the presence of others (consumers and employees) in the environment can affect consumers’ acceptance. Fourth, exploring how hotel employees can better cooperate with service robots is valuable for optimizing the human–robot interaction experience of hotel users. Fifth, further research can explore the comparative analysis of service robots with distinct functions and shapes, or the influence of interaction between diverse types of robots on consumers’ perception or experience. Finally, through a combination of multiple software and methods, future research can adopt other models and scales related to user’s willingness to accept or use service robot and compare the applicability of different models and scales to obtain more comprehensive and specific conclusions.

## Data availability statement

The original contributions presented in the study are included in the article/supplementary material, further inquiries can be directed to the corresponding author.

## Author contributions

CC: contributed to the conceptualization, methodology, statistical analysis, data curation, and writing. BS: contributed to the revision, investigation, supervision, funding acquisition, and project administration. YL: contributed to the revision. YZ: contributed to the funding acquisition, and project administration. All authors contributed to the article and approved the submitted version.

## Funding

We acknowledge the financial support from the National Natural Science Foundation of China (grant nos.: 72110107002 and 71974021) and the National Social Science Foundation of China (grant no.: 21BGL246).

## Conflict of interest

The authors declare that the research was conducted in the absence of any commercial or financial relationships that could be construed as a potential conflict of interest.

## Publisher’s note

All claims expressed in this article are solely those of the authors and do not necessarily represent those of their affiliated organizations, or those of the publisher, the editors and the reviewers. Any product that may be evaluated in this article, or claim that may be made by its manufacturer, is not guaranteed or endorsed by the publisher.
